# Different effects of hyperlipidic diets in human lactation and adulthood: growth versus the development of obesity

**DOI:** 10.1186/1477-7827-9-101

**Published:** 2011-07-28

**Authors:** Marià Alemany

**Affiliations:** 1 Department of Nutrition and Food Science, Faculty of Biology, University of Barcelona, Barcelona, Spain; 2 CIBER Nutrition and Obesity, Institute of Health Carlos III, Spain

## Abstract

After birth, the body shifts from glucose as primary energy substrate to milk-derived fats, with sugars from lactose taking a secondary place. At weaning, glucose recovers its primogeniture and dietary fat role decreases. In spite of human temporary adaptation to a high-fat (and sugars and protein) diet during lactation, the ability to thrive on this type of diet is lost irreversibly after weaning. We could not revert too the lactating period metabolic setting because of different proportions of brain/muscle metabolism in the total energy budget, lower thermogenesis needs and capabilities, and absence of significant growth in adults. A key reason for change was the limited availability of foods with high energy content at weaning and during the whole adult life of our ancestors, which physiological adaptations remain practically unchanged in our present-day bodies. Humans have evolved to survive with relatively poor diets interspersed by bouts of scarcity and abundance. Today diets in many societies are largely made up from choice foods, responding to our deeply ingrained desire for fats, protein, sugars, salt etc. Consequently our diets are not well adjusted to our physiological needs/adaptations but mainly to our tastes (another adaptation to periodic scarcity), and thus are rich in energy roughly comparable to milk. However, most adult humans cannot process the food ingested in excess because our cortical-derived craving overrides the mechanisms controlling appetite. This is produced not because we lack the biochemical mechanisms to use this energy, but because we are unprepared for excess, and wholly adapted to survive scarcity. The thrifty mechanisms compound the effects of excess nutrients and damage the control of energy metabolism, developing a pathologic state. As a consequence, an overflow of energy is generated and the disease of plenty develops.

## Background

### Lactation and the dietary induction of the metabolic syndrome

It seems crystal clear that the triggering factor of the metabolic syndrome and related diseases is our inability to process -for years- an excess of ingested energy, mainly lipid. Along our evolutionary development we have not needed -so far- to develop control mechanisms for lipid handling, since they are scarce in the environment [1], and thus of limited quantitative importance in our early-times diet. In our food energy-handling metabolism, fats are practically absent in our life *in utero *but are a main source of energy during lactation because of its substantial presence in milk, decreasing sharply after weaning.

The main role of lipids, from infancy and throughout all our adult life, is limited to constitute the mainstay of our own energy reserves. Consequently, massive mobilization of body fat reserves in adulthood is a strong indication (in metabolic terms) that our energy homeostasis is in jeopardy, and thus we ought to adopt a survival mode [2,3]. However, during lactation, lipid (and sugars: lactose) are -precisely- our main sources of energy, the rest being essentially high-quality protein [4] minerals and microcomponents. Everything is used both for growth & development and, secondarily, to maintain thermogenesis.

It is both curious and telling how the pattern of nutrient intake of a high excess of energy in adulthood has a more than casual resemblance with lactation in our early infancy. The coincidence is perhaps even greater because of the choice of foods in present-day adult diets, in which dairy products play a significant role.

We are born fully prepared for milk, a lipid-and-disaccharide-based hyperproteic diet. Milk mimics the diet that cause the metabolic syndrome in adulthood: the main carbohydrate is a dissaccharide, the main source of energy is lipid, also rich in saturated fat and cholesterol, and the protein is largely high-quality, with a remarkable proportion of essential amino acids; milk also contains a sizeable proportion of minerals (calcium, phosphate), and water. This complete diet is intended to help us grow fast for a limited time [5] at the expense of the mother's ability to transform low quality foods into the very rich mixture that constitutes milk. Shortly afterwards, when the lactation period ends, our body metabolism shifts from lipid-based energy to carbohydrate and low-quality protein during the weaning period [6], adopting the adult stance and implementing irreversible metabolic adaptations that will last for life.

The use of fats as main food is a shift from intrauterine life, in which they are practically not used for energy (its ability to cross the placenta is limited), but stored (*baby fat*) after being synthesized from maternally-provided glucose [7]. The fetus grows using blood-borne transplacental amino acids and its energy needs are essentially sustained by glucose [8]. Immediately after birth, the need to maintain body temperature, largely through thermogenesis, suddenly develops [9], and consequently, the need for energy skyrockets. Only dietary fats can supply packed energy in a sufficient, safe and effective way, in comparison with sugars (and the large amounts of water needed to transfer them without dangerous osmotic consequences), allowing also for a fast growth. This situation continues just until the newborn is able to mimic the feeding habits of the mother, and her ability to supply the growing demands of milk energy levels off.

### The metabolic shift of weaning

In most mammals, weaning is an irreversible process, which is biochemically established by the practical loss of the brain ability to use ketone bodies [10] and the disappearance of intestinal lactase [11], parallel to the full functionality of the Leloir pathway [12] a shift in the composition of our intestinal microbiota [13], a better retention of minerals and essential amino acids [14], and a slower rate of growth [15] as main traits. Perhaps the key change is the shift from a system based on lipolysis and fatty acid oxidation to a system based on carbohydrates, centered on glycolysis, and the derivation of excess acetyl-CoA towards lipogenesis [16]. In both cases, the main provider of energy is acetyl-CoA, but the regulation of 2C and 3C metabolism establishes a definitive pattern that extends to the whole body with organ maturation [17]

Weaning rats to a standard low-energy rat chow and later exposing them to a high energy diet (cafeteria diet), we can observe widely different effects. The animals eventually develop obesity [18,19], but it takes some time because the adult mode of lipid handling had been already set in. In a way, the development of the metabolic syndrome in adulthood, after several years of exposure to excess lipids resembles the pre-weaning situation: lipids are the mainstay of the body energy economy.

However, the reversion to lactation of an adult poses serious additional problems. The composition of the diet is not a critical aspect; in fact, the abundance of dairy products themselves combined with the nutrients (high quality protein, fats and sugars) present in our diets make them comparable to the nutrient composition of milk. The main problems are those derived from the role of glucose in the regulation of energy distribution between tissues [20], the maturation and growth of energy needs (glucose) of the brain [21], the role of muscle (limited in small children, but critical in adulthood) and adipose tissue, a store of energy in the baby, but also an organ implied in the maintenance of energy availability in the adult [22-24]. The differences extend to the full setting and operativiteness of steroid hormones [25,26] and the control of body functions by the autonomic nervous system. The different quantitative role of thermogenesis, which takes up a large chunk of energy in the newborn [27], but little in most adults [28] under normal conditions, also helps change the picture. And last, but not least, the adult body is geared to retain essential amino acids and amino N in general (theoretically scarce)[29], using part of the dietary protein to obtain energy. The baby is designed to use most of the available dietary protein for growth, to sustain an active synthesis of protein [30], and accepts a certain degree of inefficiency [31] (at the expense of the mother's substrate-providing altruism). This could not be the case for an adult, in which strict N conservation schemes have been established [32].

**Figure 1:**
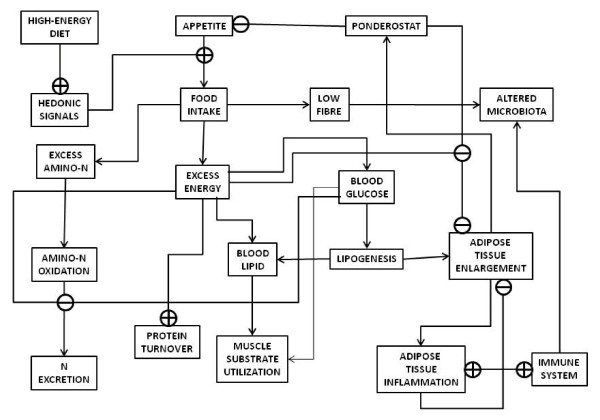
**Diagram of the main metabolic consequences of the continued ingestion of a diet containing excess energy, with abundant lipid, protein and carbohydrate**.

The prolonged lipid exposure after weaning the rat pups using a cafeteria diet (the same consumed by their dams) results in a rapid and continued growth that soon shifts into obesity and a metabolic syndrome-style derangement [18,19]. In fact, milk composition is also altered by exposure of the dam to a lipid-enriched diet [32,33]. These effects help us to differentiate which are the factors eliciting the metabolic syndrome under a milk-like diet in adulthood (Figure 1):

1. Loss of the brain ability to adjust energy intake to energy needs (or vice-versa), mainly because the cortical hedonic signals seem to override the basic ponderostat signaling on appetite, compounded by the loss of importance of thermogenesis (depending on body size) as main energy controlling element in the adult [34,35] as compared with newborns. This includes changes in the regulation and secretion of neurotransmitters [36], the control of glucocorticoids [37] and other nervous system-regulated defense mechanisms, essentially through the autonomous nervous system [38].

2. Conflict between high availability of amino acids and the genetically ingrained drive to preserve amino N, and, especially, essential amino acids, as in starvation [39,40]; this results in altered N excretion mechanisms and patterns [41].

3. Loss of the primacy of glucose as main inter-organ energy transporter, and alteration of its control because of interfering fatty acids [42]. Muscle massive use of lipid in detriment of glucose generates a sustained excess of unused glucose [43].

4. Alteration of the function of adipose tissue towards a more passive role of (excess) energy storage rather than its active control of energy availability [44]; this is a direct consequence of the decrease in brain control (point 1). Excess lipid storage causes a lipotoxic or inflammatory situation that plays havoc with energy control mechanisms (points 2 and 3) [45]. This implies the direct intervention of the immune system in the defense against excess energy accumulation and uncontrolled growth of adipose tissue [46,47].

5. In humans, deep alteration of the microbiota composition, and of our mutualistic relationship with it [48], via alteration of NO_X _excretion [49,50], shift to lipid abundance [51], lower fiber and complex carbohydrate availability, and increased immune reactivity (changes in intestinal barrier defense function and strategies) [52,53]. This is a consequence of points 2, 3 and 4 in addition to dietary alteration.

6. As a consequence of brain and adipose tissue signaling changes (points 1 and 3), regulation of protein turnover (points 2, 3 and 4) [54], protection against oxidative aggression [55] and other maintenance functions provided by androgens and estrogens [56] are also altered, resulting in an imbalance in the equilibrium between their molecular forms that aggravates the basic damage of diet alone

The metabolic situation in an adult receiving an excess energy diet, with a nutrient composition not too much different from that of milk is different from that of sucklings. Now there is no need of an extra galactose supply for brain galactolipid synthesis, since it is synthesized -on demand- from UDP-glucose [12]. In adults, excess dietary galactose eventually enters the glycolytic pathway. However, high spikes of circulating glucose induce an insulin response that may help develop insulin resistance compounded by the increased -and preferential- muscle consumption of lipid [57]. In addition, large loads of galactose (or fructose) misadjust the control mechanisms of monosaccharide transport and metabolism, set for glucose [58]. The shift to glucose as main energy staple is already completed during early infancy, and the brain cannot use ketone bodies in significant proportions [59]. There is a problem of excess glucose; the amino acid needs for growth decrease, and their catabolism to urea is hampered [41]; 2-amino nitrogen excess becomes a nuance.

A few of the mechanisms used during our milk-only suckling period are put in action again (at least to a limited extent) in adult overfeeding: increased thermogenesis [60] and protein turnover [61], use of fatty acids as main energy substrate [62], preservation of glucose, essential amino acids and amino N [63], and enhancement of the independence and capabilities of the defense (immune) system [64]; but the rest of conditions are different: the brain is already fully functional, muscle is active, comparatively larger and mature, the importance of thermogenesis is limited [65], and -especially-, growth is arrested; the tolerance of brain to ketone bodies and acidosis is lower than during development, glucose homeostasis is more regulated, and brain cortical influences (i.e. over appetite and food selection) are again different (more focused and powerful), often overriding the automatic mechanisms controlling appetite and food intake [35]. The old recipes do not work well for the new situation.

### Conditions for the development of the disease of plenty

The disease of plenty is a wider definition of the metabolic syndrome encompassing a large number of common-origin diseases expressed in parallel to the metabolic syndrome (it is a disease that requires a long time of development and the confluence of a series of internal and external events to manifest, we can try to define a sequence of events that finally result in the full manifestation of this disease in adulthood.

The sequence of development of the metabolic syndrome starts with:

1 A more than probable genetic [66-68] and/or epigenetic [69,70] upbringing (i.e. allele natural selection, activation of thrifty genes) [67,71], starting in the womb [72,73] (or even in prior generations [74,75]), set the conditions for optimal use of energy resources along the whole ensuing lifespan. This is applicable, especially to populations that have access to abundant choice (high-energy, highly palatable) food resources following a previous period of limited nutrient availability, famine, man-made scarcity or alimentary monotony [76,77]. The growth of the proportion of obese in developing nations agrees with this interpretation [78,79]. There is a wide consensus to explain this situation in the existence of thrifty genotypes for isolated populations (*survivors' genes*) [80], completed by recent studies of trans-generational transfer of information on energy availability via epigenetic modification of our final functioning genome [81,82].

2 Full availability of food, varied food, in which the proportion of high quality protein (meats, dairy) is concurrent with sweets (sugars, fruit) and large proportions of lipid (oil, lard, tallow, greasy meats, nuts) and salt. The appetite for these historically scarce (and widely desired) materials is ingrained in our brain in an atavistic way [83], comparable perhaps to a number of phobias (spiders, snakes) that we also inherited from our early ancestors. Unlike protein [84,85] (and largely sugars [86,87] we have not developed mechanisms controlling the eventual excessive intake of lipid. Consequently, our ancestors of less than a hundred generations, ate no more than 20% of their energy needs as lipids, and about 10-12% protein (mostly low-quality and plant-derived), with a limited contribution of sugars (fruit) and no added salt (except in seafaring cultures) [88]. The recommended diets of today maintain the 10-12% proportion of energy as protein (but with a significant higher share of animal- or nut-derived high quality protein), the proportion of sugars, added or present in foods (fruit, milk) may reach as much as 10% of our daily energy [89]. Fats are recommended to be consumed with a higher limit of about 35% of energy needs [90]. Low (!) salt diets contain up to 3-7 g of salt per day [91]; even hospitalized people may receive more than 6-8 times as much NaCl i.v. per day. The difficulties arise largely from the collision of our ancient genome and accompanying physiological adaptations on one side the wide shift in dietary composition and energy availability on the other [92]. Thus an efficient early human machinery, adapted to extract glucose and amino acids from complex polysaccharides mixed with fiber and low biological quality protein, has to cope with a surcharge of salt, sugar, readily hydrolyzable protein (with unnecessary abundance of essential amino acids), limited amounts of complex carbohydrate and fiber and, especially, a brutal daily overload of fat.

3 We endure a marked change in the environmental conditions we are geared for: decreased danger of being pray to predators (if we exclude lawyers and banks), lessened preoccupation for gathering food for the everyday meal, regularity of meals and the possibility of nibbling tasty food almost continuously, choice of foods, abundance of edible materials [1], but also lower need to do exercise, rampant sedentary habits [93], and the stress derived from our lack of physiological and psychological adaptation to a man-made environment of exponentially growing complexity. This applies both to technical advances and social structure changes that drastically alter (usually wiping-out) centuries-old cultural traditions in less than one generation.

These three factors combine to develop the disease of plenty in a growing proportion of the whole World population. The genetic influence is indubitable because of the evidence gathered in studying population changes [94,95] genetic composition associated to eating habits [96], linked to the incidence of genetic markers and a few diseases of metabolic origin [97]. We can add the early setting of our ponderostat and efficiency adjustments [98,99], and the growing evidence of epigenetic trans-generational adaptation [100,101]. However, no single gene or allele has proved to be the cause of mainstream obesity [102], in spite of minoritary monogenic obesity in humans [103]. For this reason there is a growing consensus that genetic contribution to obesity, the metabolic syndrome and related diseases is essentially permissive, albeit probably also necessary.

The relationship of the nature and richness (in energy and fats) of the diet as main long-term cause for obesity and related diseases is overwhelmingly accepted. However, the precise reason or mechanism explaining why this excess energy (essentially lipid energy) finds its way into our bloated bodies is the elusive subject of intense research and speculation, as proved by the growing amount of literature published on these issues [104,105]. Notwithstanding, and irrespective of having the main culprit well identified, dietetic counsel to the population continues following the dictates of the Food Industry rather than the scientific evidence so far available on this subject [106]: we keep gorging on large amounts of fats, salty, sugary and protein-laden savory foods [107,108]. A few do a Copernican somersault and revert to also unhealthy natural or organic foods, adopting highly restrictive diets; they pervert the meaning of natural food, since even the most elaborate industrial foods are made of natural ingredients, and all of them are of organic nature with the obvious exceptions of water and minerals. However, the people subject to vegan or strictly natural diets seldom develop obesity and related diseases [109]; they may, instead, develop nutritional deficits due to their inadequate diets [110,111].

Finally, sedentary behavior, lack of motivated exercise (there is no need to search for food or to flee from predators) affects a large part of our population [112]. This is especially true of workers, which tend to shift from agriculture and industry (both now largely mechanized and dependent on power tools, not on muscle power) to services, the paradigm of sedentary work. Mobility is left to mechanical devices; most chores are not dependent on muscle anymore, and exercise for health or aesthetics is a remedial substitute that is practiced by only part of the society [113]. Stress is a powerful signal inducing the alteration of appetite [114]; it also enhances glucocorticoid-induced insulin resistance and fat accumulation [115]; but our ancestors did not live without stress, and we are endowed for sudden, intense and life-threatening situations of fight-or-flee. However, we are ill-equipped for continued low-level stress, such as the universal worrying for economic and sentimental questions or the unnatural invasion of our personal space in public places [116].

## Conclusions

In spite of human temporary adaptation to a high-fat (and sugars and protein) diet for a critical growth and development period -lactation-, after weaning and the end of development into adulthood the ability to thrive on this type of diet is lost. The main reasons for that change are essentially linked to the historically limited availability of high-energy packing foods at weaning and during the whole adult life of humans. Thus our bodies have been adapted for many generations to survive with relatively poor diets interspersed by bouts of scarcity and plentifulness (unpublished). Present-day diets, made up from our deeply ingrained food desire for fats, protein, sugars, salt and hedonic components of food, are markedly rich in energy and these same components listed [1], similar in nutrient proportions to a milk-like diet. However, most adult humans are wholly unprepared for these types of diet and cannot process the excess food ingested because our cortical-derived desire overrides the mechanisms controlling appetite. As a consequence, an excess of energy is generated and the disease of plenty develops. This is produced not because we lack the biochemical mechanisms to use this energy, but because we are unprepared for excess and wholly adapted to survive scarcity; these same mechanisms compound the effects of excess nutrients and damage our mechanisms of control of metabolism, developing pathologic states. We could not revert too the lactating period metabolic setting because of different proportions of brain/muscle metabolism, lower thermogenesis needs and capabilities and absence of significant growth in adults

## Competing interests

The author declares that they have no competing interests.
